# Impact of different genomic relationship matrix construction methods on the accuracy of genomic prediction in different species

**DOI:** 10.3389/fgene.2025.1576248

**Published:** 2025-05-02

**Authors:** Shiyi Wang, Yingjia Wei, Dengying Liu, Xiangzhe Zhang, Qishan Wang, Yuchun Pan, Peipei Ma

**Affiliations:** ^1^ Shanghai Collaborative Innovation Center of Agri-Seeds/School of Agriculture and Biology, Shanghai Jiao Tong University, Shanghai, China; ^2^ Department of Animal Breeding and Reproduction, College of Animal Science, Zhejiang University, Hangzhou, China

**Keywords:** genomic relationship matrix, accuracy of prediction, different species, size of reference population, marker density

## Abstract

**Objective:**

Genomic best linear unbiased prediction (GBLUP) is a key method in genomic prediction, relying on the construction of a genomic relationship matrix (G-matrix). Although various methods for G-matrix construction have been proposed, the performance of these methods across different species has not been thoroughly compared.

**Methods:**

This study systematically evaluated the performance of six genomic relationship matrix (G-matrix) construction methods in improving the prediction accuracy of GBLUP models across four species: pigs, bulls, wheat, and mice. The methodological framework included: (1) an initial unscaled matrix; (2) five scaled methods utilizing allele frequency centralization. The scaled methods comprised: (a) three variance-weighted approaches using allele frequencies fixed at 0.5 (G05), observed frequencies (GOF), or average minor allele frequencies (GMF); (b) two centralized methods with weighting by either the trace of the numerator matrix (GN) or reciprocals of each locus’s expected variance (GD).

**Results:**

The GD matrix demonstrated significant prediction accuracy improvements for pig traits. Conversely, most scaled G-matrices showed minimal effects on mice, wheat, and bull, even with underperforming unscaled baselines in prediction accuracy compared to the original unscaled matrix. The learning curve for bull data showed the choice of G-matrix had minimal impact on prediction accuracy when the reference population size and genetic marker density reached a certain threshold.

**Discussion:**

The study concluded that the optimal G-matrix construction method varies across species, with population structure being a key factor. These findings highlight the importance of species-specific optimization in genomic prediction and suggest that the influence of G-matrix construction diminishes in large-scale, high-density genomic datasets.

## 1 Introduction

Genomic prediction (**GP**) is a method of genetic evaluation of individuals using information from genome-wide genetic markers (Desta and Ortiz, 2014). With the rapid development of sequencing technology (Slatko et al., 2018), the cost of high-throughput single nucleotide polymorphism (**SNP**) marker detection continues to decrease. GP is widely used in animal and plant breeding practices nowadays. Compared with conventional breeding approaches, this method offers significant advantages, including shorter generation intervals, higher predictive accuracy, and reduced operational costs (Zhang et al., 2010). Various models have been used for genomic prediction. Bayesian variable selection models are generally more accurate than genomic best linear unbiased prediction (**GBLUP**) in predicting genomic breeding values (VanRaden et al., 2009). However, GBLUP is generally used for routine genomic evaluations because of lower computation. Compared with traditional BLUP (Momen and Morota, 2018), the genomic matrix (**G-**matrix) of GBLUP can reflect differences in genetic information between individuals and reduce the deviation caused by Mendelian sampling (El-Kassaby et al., 2012). The accuracy of predicting breeding values using genomic data is significantly higher than that when using genealogical records (Jonas and de Koning, 2015).

The G-matrix could be constructed simply by multiplying genotype matrix M and its transpose matrix M′ to count the number of alleles shared by relatives. This method was directly derived according to the properties of variance. Several methods were then proposed to modify the G-matrix to make it comparable to the A-matrix (Wright, 1922). A normalized matrix (**GN**) ensured that the average diagonal element was close to 1. In other methods, the allelic frequency from the base population was needed in constructing **G** to increase the weight of rare alleles. However, it was not available since those individuals in the base population were not genotyped in reality. Therefore, researchers have proposed a variety of alternative approaches to obtain the frequencies: 0.5 for all markers (**G05**), the average minor allele frequency (**GMF**), or the observed allele frequency of each SNP (**GOF**). The covariance matrix of additive genetic effects is generally defined as proportional to G in GBLUP. The four previously described G-matrices assumed that all markers contribute equally to the genetic variation of the same trait. This assumption is not desirable if the trait is influenced by major genes. To address this limitation, the **GD** matrix weight markers are evaluated by reciprocals of their expected variance instead of applying uniform scaling across all loci (VanRaden, 2008). The efficiencies of different G-matrices were compared in a previous study, which mainly aimed to identify the optimal method for constructing the G-matrix in single-step BLUP. The mean and variance of the diagonal and off-diagonal elements, the variance of genotyped individuals, and the accuracy of genotyped female individuals varied among the different G-matrices. The authors recommended using the GN matrix in single-step BLUP since it was the most compatible matrix with the A-matrix. G05 does not require the frequency of the second allele, which is suitable for the situation where the total population or the genotype of some individuals is unknown. GMF is similar to G05, except that 0.5 is replaced by the mean value of the frequency, which is suitable for the base population when some allele frequencies are unknown. When the average inbreeding coefficient is low or the number of generations is small, the mean value of the diagonal and off-diagonal elements is greater than the coefficient in the pedigree in G05 or GMF. On the contrary, the mean of the diagonal elements in GOF will be smaller than the coefficient in the pedigree, and the mean of the off-diagonal elements is 0. GOF is currently the most widely used G-matrix. If the amount of data is small, a G-matrix with an average diagonal coefficient not equal to 1 will result in a large additive variance. The normalized GN matrix can reduce the additive variance. GN can better correspond to the pedigree matrix (A-matrix) when pedigree information is needed and the inbreeding coefficient is low. GD has a higher pertinence to the trait affected by major genes. The calculation process of GD is relatively complex, but it is more effective in researching human genetic diseases (Amin et al., 2007) compared with GOF and GMF. However, how different G-matrices performed in GBLUP and which method was robust in different species were not investigated in previous studies.

The objective of this study was to compare the impacts of six G matrices on the accuracy of GBLUP. Four different species were used, namely, pigs, mice, wheat, and cattle, which also differed in population size and the number of genetic markers.

## 2 Materials and methods

### 2.1 Data

Four different species were used in the study, namely, pig, wheat, mice, and bull. Details of the data are shown in Table 1.

**TABLE 1:** Summary of datasets.

Species	Number of individuals	Number of genetic markers
Pig	820	44,580
Mice	1,814	10,346
Wheat	599	1,279
Bull	5,024	42,551

There were 820 commercial female pigs from a published study (Fan et al., 2011), which were genotyped using the Illumina PorcineSNP60 BeadChip. After quality control, 44,580 SNPs remained. Phenotypic traits comprised the 10th rib backfat (**bf10**), last rib backfat (**lastrib**), and loin muscle area at the 10th rib (**LMA**).

Mice and wheat datasets were obtained from the BGLR reference manual (https://cran.r-project.org/web/packages/BGLR/BGLR.pdf) of R software (de los Campos et al., 2009). The mice dataset was composed of 1,814 individuals, each genotyped for 10,346 polymorphic markers. The traits presented here were body mass index (**Obesity_BMI**), body weight (**EndNormalBW**), and body length (**Obesity_BodyLength**). The wheat dataset was from the Global Wheat Program of the International Maize and Wheat Improvement Center (**CIMMYT**), Mexico. Information was collected from 599 historical CIMMYT wheat lines. Wheat lines were genotyped using 1,447 Diversity Arrays Technology (**DArT**) generated by Triticarte Pty., Ltd. The number of DArT MMs after editing was 1,279. The environments in the trials were grouped into four typical agro-climatic regions, and the trait analyzed was the average grain yield (**GY**) of 599 wheat lines in each of these four mega-environments.

Bull data were derived from the study by Zhang et al. (2015), which used a German Holstein population of 5,024 bulls provided by Vereinigte Informationssysteme Tierhaltung w.V. (Zhang et al., 2015). All bulls were genotyped using the Illumina BovineSNP50 BeadChip (Matukumalli et al., 2009), and 42,551 SNPs remained after quality control. The three traits were milk fat percentage (**FP**), milk yield (**MY**), and somatic cell score (**SCS**), which had highly reliable conventional estimated breeding values. They may represent three genetic structures of complex traits, namely, one major gene and many small effect loci (**FP**), few moderate effect loci and many small effect loci (**MY**), and many small effect loci (**SCS**) (Hu et al., 2013; Zhang et al., 2014).

Genetic markers were screened by deleting SNPs with a minor allele frequency (**MAF**) less than 0.05.

### 2.2 GBLUP statistic model

The GBLUP model used in this study was as follows:

y=Xb+Zg+e,

where y is the phenotypic vector; b is the fixed effect vector; X is the design matrix for b ; g is the random additive genetic effect vector following a normal distribution $N\left(0,G\sigma_{g}^{2}\right)$ ; G is the genomic relationship matrix; $\sigma_{g}^{2}$ is the genomic additive variance; Z is the design matrix for g ; and e is the random residual, which obeyed the normal distribution with mean **0** and variance $I\sigma_{e}^{2}$ .

### 2.3 Genomic relationship matrix

The G-matrix was constructed based on genetic markers. This study compares the effects of six G-matrix construction methods on the accuracy of GBLUP.

SNP information was transformed into a digital matrix containing the number of minor alleles (**M** matrix), represented by 0, 1, and 2, respectively. The number of rows in M is the number of individuals (*n*), and the number of columns is the number of markers (*m*). M matrix times its transpose is MM′, which is the first method of constructing the G-matrix.

To set the mean values of the allele effects to 0, M was modified by subtracting 2 $p_{i}$ , where $p_{i}$ is the frequency of the second allele at locus *i*. Each column *i* of matrix P corresponds to 2 $p_{i}$ . In addition, $2\sum p_{i}\left(1-p_{i}\right)$ was used as the denominator to scale the G-matrix, making it comparable to the A-matrix. The formula can be expressed as follows:

$$G=\frac{{\left(M-P\right)\left(M-P\right)}^{\prime }}{2\sum p_{i}\left(1-p_{i}\right)}.$$

Allele frequencies should ideally be obtained from the unselected base population, but this was not available. Instead, these frequencies were expressed as 0.5 for all markers (**G05**), the average minor allele frequency (**GMF**), and the observed allele frequency of each SNP (**GOF**).

In the GN and GD method, $p_{i}$ in matrix P is the observed allele frequency of each SNP.

G is normalized in the GN method. We obtain the normalized GN by dividing by the mean of the trace of ${\left(M-P\right)\left(M-P\right)}^{\prime }$ as follows:

GN=M−PM−P′traceM−PM−P′n.

For the GD method, the diagonal D-matrix is used to add weight to markers. D is a diagonal matrix in which all off-diagonal elements are 0, and the diagonal elements are calculated as follows:

$$D_{ii}=\frac{1}{m\left[2p_{i}\left(1-p_{i}\right)\right]}.$$

GD modifies itself using the inverse of the markers’ expected variances rather than relying on the sum of expectations across multiple loci.

$$\text{GD}=\left(M-P\right)D{\left(M-P\right)}^{\prime }.$$

### 2.4 Cross-validation

K-fold cross-validation was used to test the accuracy of genomic prediction using different G-matrices. First, the data were divided into k folds. One fold was used as the test set each time, and the other folds were used as the training set. The training set was used to build the association between genetic markers and phenotypes. The test set was used to predict the phenotype based on the constructed model and compare it with real observations to verify the accuracy of genomic prediction.

For cross-validation, pigs and wheat were divided into 10 groups for phenotypic value prediction. Mice were randomly divided into 15 groups, and bulls were randomly divided into 32 groups. Notably, the number of individuals in one group in the wheat and mouse datasets was one less than the other groups, which did not affect the results (Wang et al., 2017).

GBLUP analysis is performed using self-coded scripts running in R software (R 4.1.1). The result analysis mainly refers to $R^{2}$ (coefficient of determination), which was used to measure the goodness of fit. The closer the value of $R^{2}$ is to 1, the better the regression line fits the observed data. The calculation formula of R^2^ is as follows:

$$R^{2}=1-\frac{PRESS}{SST},$$

where PRESS is the predicted residual error sum of squares and *SST* is the sum of the squared total.

### 2.5 Learning curves with bull data

The learning curve was used to compare the performance of genomic prediction strategies (Ou and Liao, 2019). To evaluate the effects of reference population size and marker density on the accuracy of genomic prediction across different genomic relationship matrices (G-matrices), we implemented a comprehensive sampling strategy involving systematic variations in both parameters.

We examined seven levels of reference population size (500, 1,000, 1,500, 2,000, 2,500, 3,000, and 4,000 bulls), with each size level independently replicated six times through random sampling to ensure robust estimates of prediction accuracy.

For the marker density analysis, we fixed the reference population size at 5,024 bulls and created 5 geometrically spaced density levels by subsampling from all available markers, specifically analyzing milk yield as our target trait. These density levels were generated by sampling one SNP per 16 markers (yielding 2,660 SNPs), per 8 markers (5,319 SNPs), per 4 markers (10,638 SNPs), and per 2 markers (21,276 SNPs) while maintaining consistent genomic distribution and quality control standards across all subsets.

All the scenarios used 10-fold cross-validation.

## 3 Results

### 3.1 Accuracy of phenotype prediction in pigs

**FIGURE 1:**
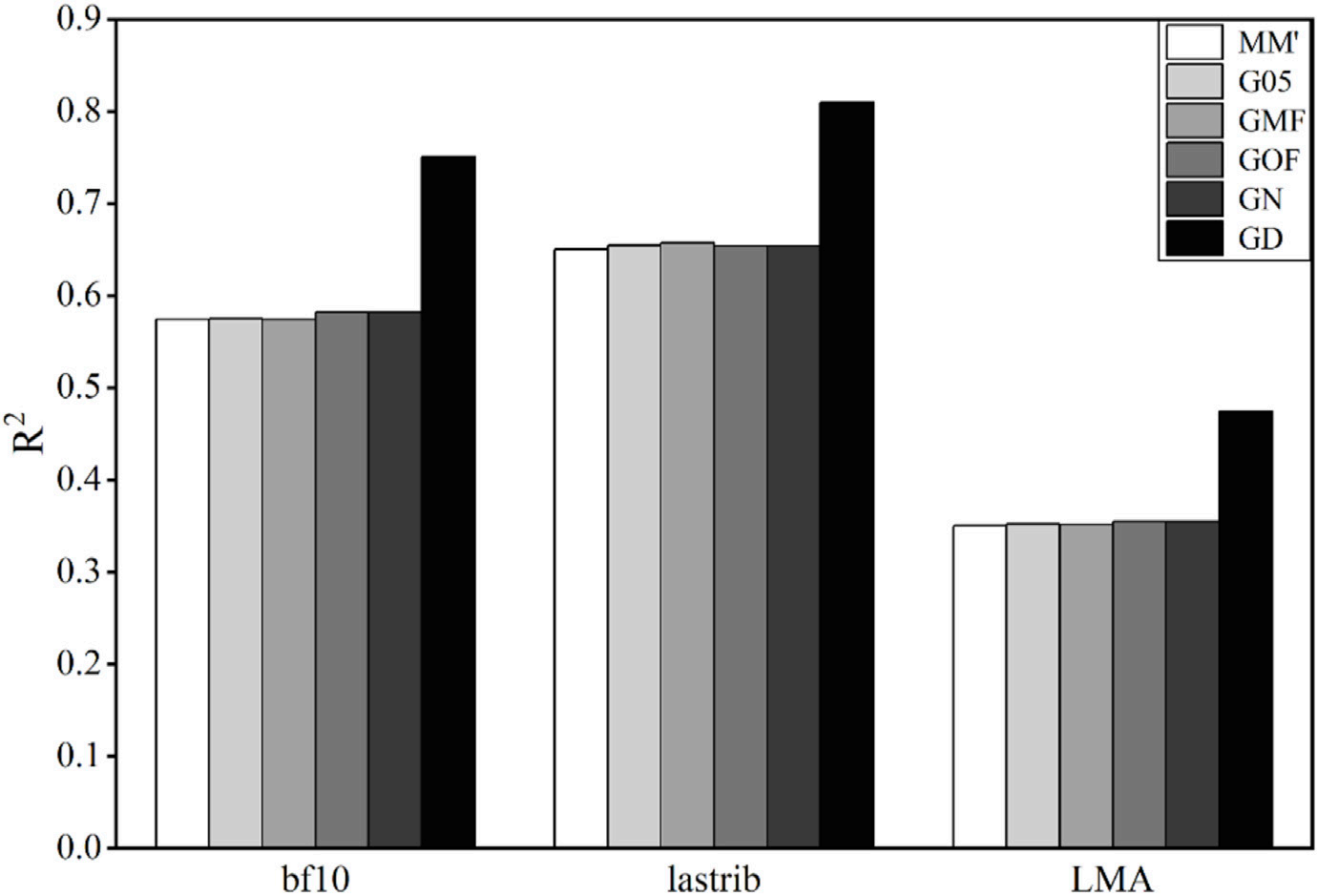
Coefficient of determination ( $R^{2}$ ) using different G-matrices in pig data. bf 10, 10^th^ rib backfat of the pig; lastrib, last rib backfat of the pig; LMA, loin muscle area at the 10^th^ rib of the pig.

The coefficients of determination ( $R^{2}$ ) for using different G-matrices in pig data are presented in Figure 1. For all three traits, GD obtained the highest $R^{2}$ value (0.751 for bf10, 0.810 for lastrib, and 0.474 for LMA). MM′ achieved the lowest $R^{2}$ value, equaling 0.574. Compared with MM′, G05, GMF, GOF, and GN all increase $R^{2}$ by less than 0.01, which is not obvious. The results of lastrib and LMA prediction are similar to those of bf10. The difference between values of $R^{2}$ obtained by G05, GMF, GOF, and GN and that obtained by MM′ is less than 0.01. Meanwhile, the $R^{2}$ value obtained by GD is significantly greater than that of MM′, and the difference between them for lastrib and LMA was 0.16 and 0.12, respectively. The prediction accuracies of bf10 and lastrib are higher than that of LMA in general.

### 3.2 Accuracy of phenotype prediction in mice

**FIGURE 2:**
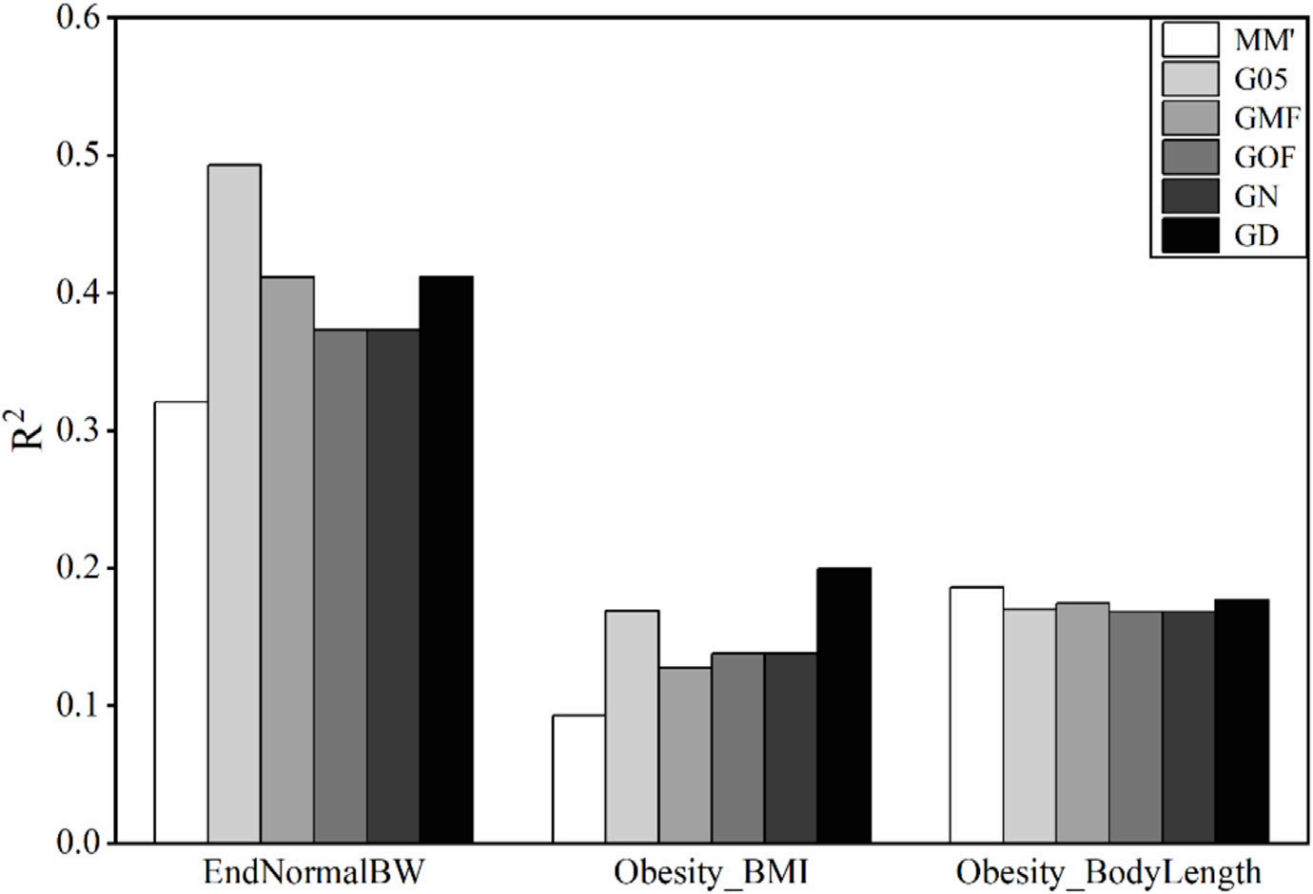
Coefficient of determination ( $R^{2}$ ) using different G-matrices in mice data. Obesity_BMI, body mass index; EndNormalBW, body weight; Obesity_BodyLength, body length. Obesity_BMI and Obesity_BodyLength both had low values of $R^{2}$ , less than 0.2. The value of $R^{2}$ of Obesity_BW is slightly higher than that of the other two but not more than 0.5. In general, the accuracy of the GBLUP model in predicting Obesity_BMI, Obesity_BL, and Obesity_BW of mice was low. Values of $R^{2}$ of Obesity_BL predicted by six types of G-matrices are almost the same, which is approximately 0.17. For values of $R^{2}$ predicted by Obesity_BW and Obesity_BMI, G05 and GD were significantly higher than MM′. GOF and GN obtained similar accuracy for all these three traits.

Six methods were used to construct the G-matrix to predict obesity correlation traits of mice. The results of the model accuracies using these six matrices are shown in Figure 2.

### 3.3 Accuracy of phenotype prediction in wheat lines

**FIGURE 3:**
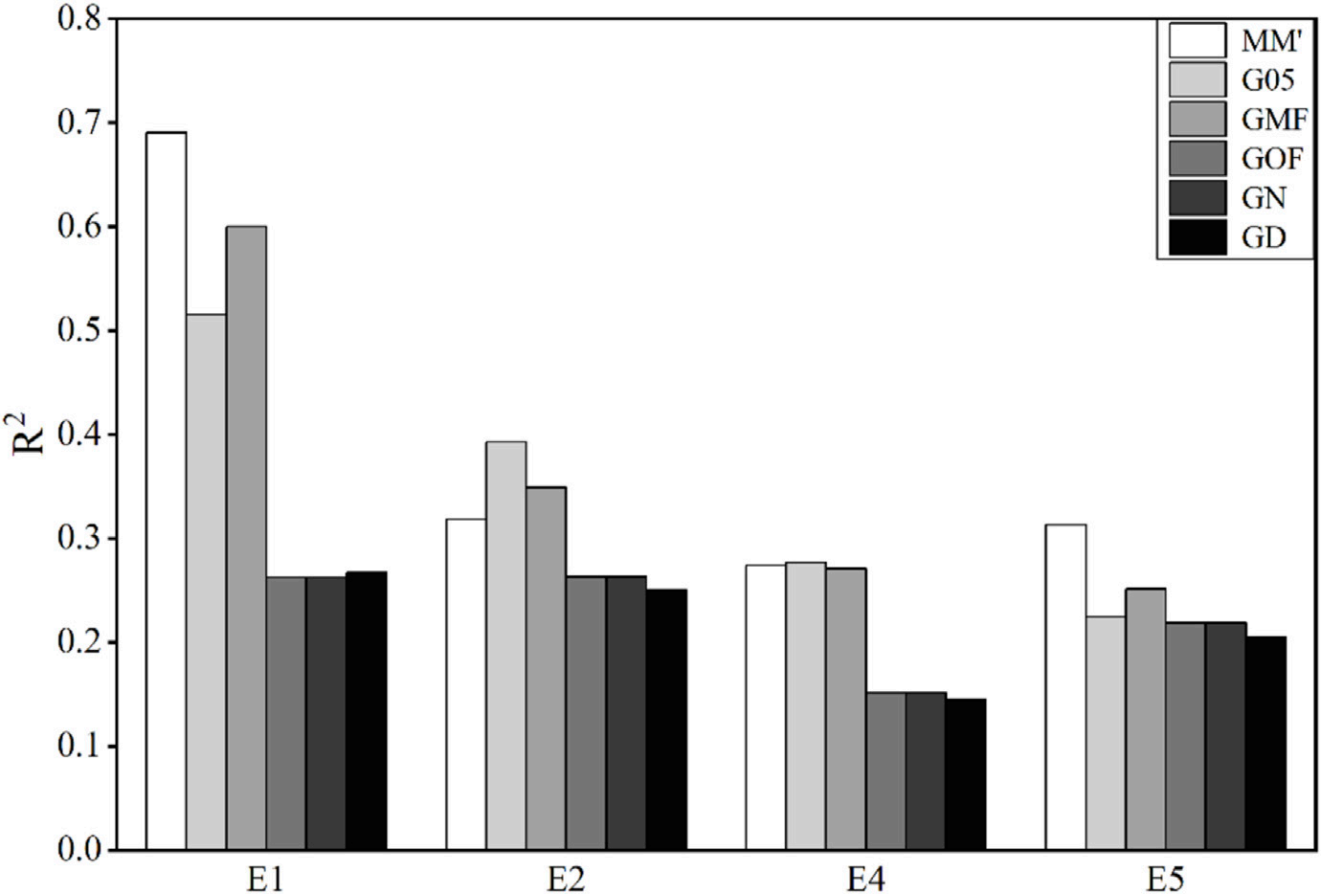
Coefficient of determination ( $R^{2}$ ) using different G-matrices in the wheat data. E means environment.

The traits of wheat used in this study were the average grain yield (GY) in four main agro-climatic regions. The accuracies of genomic prediction using these six matrices are shown in Figure 3.

The accuracies of prediction of GY in E2, E4, and E5 environments are very low, and all values of $R^{2}$ fluctuate between 0.1 and 0.4. Values of $R^{2}$ of GY predicted by MM′, G05, and GMF in E1 exceed 0.5, especially the value of $R^{2}$ obtained by MM′, which reaches 0.69. However, the values of $R^{2}$ , obtained using GOF, GN, and GD to predict GY in E1, are obviously lower than those of the other three matrices. This result also applies to E2 and E4. Overall, the unscaled MM′ performs better and more consistently in GY of wheat.

### 3.4 Accuracy of phenotype prediction in bulls

**FIGURE 4:**
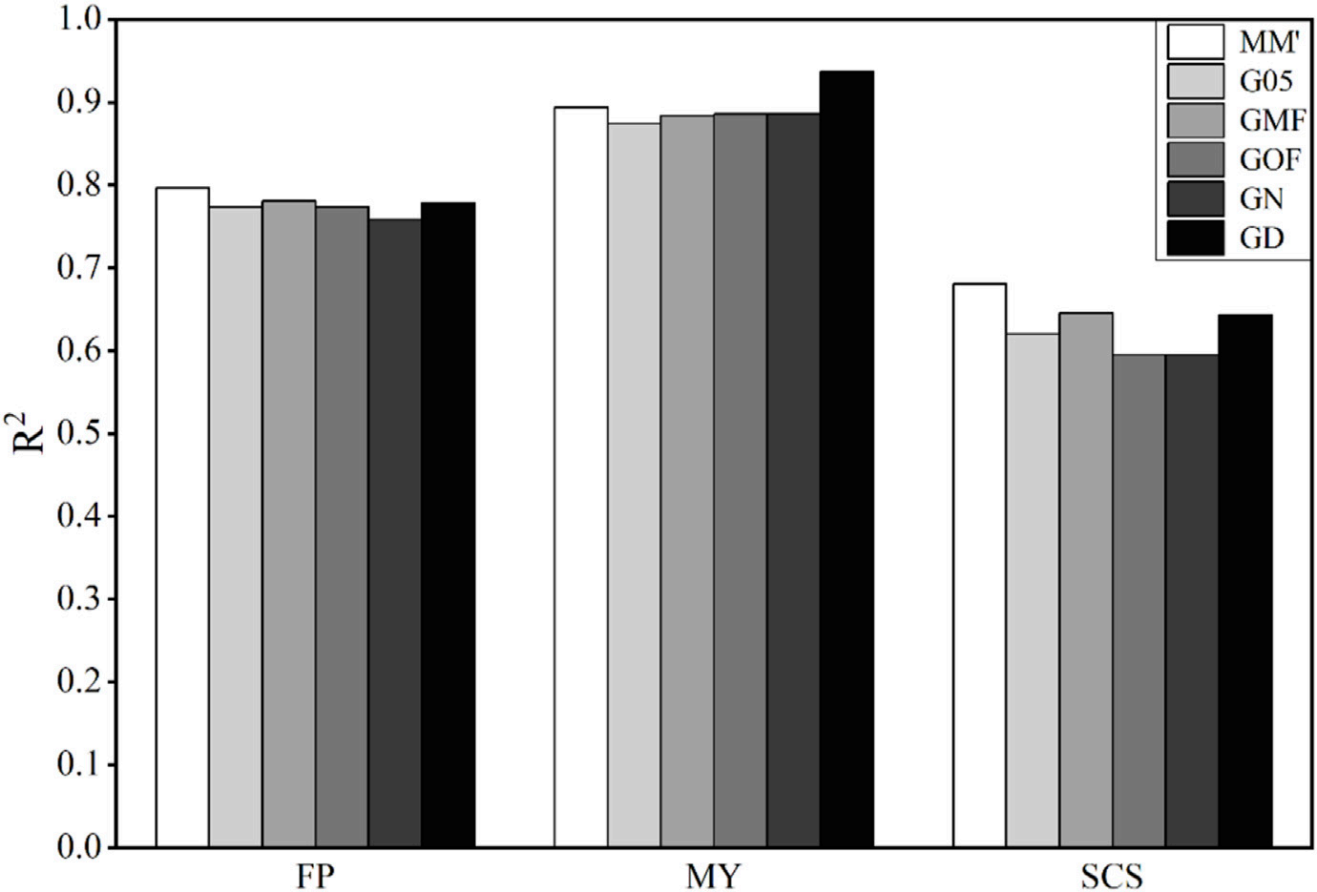
Coefficient of determination ( $R^{2}$ ) using different G-matrices in the bull data. FP, milk fat percentage; MY, milk yield; SCS, somatic cell score.

The results obtained using these six matrices to predict the traits of bulls are shown in Figure 4. Accuracies of genomic prediction for these three traits are very high. The average values of $R^{2}$ of FP, MY, and SCS are 0.630, 0.893, and 0.777, respectively. In the prediction results of MY, GD obtained the highest value of $R^{2}$ , which was 0.937. There is no apparent difference in the influence of the six matrices on the prediction accuracy of the GBLUP model.

### 3.5 Comprehensive comparison

**FIGURE 5:**
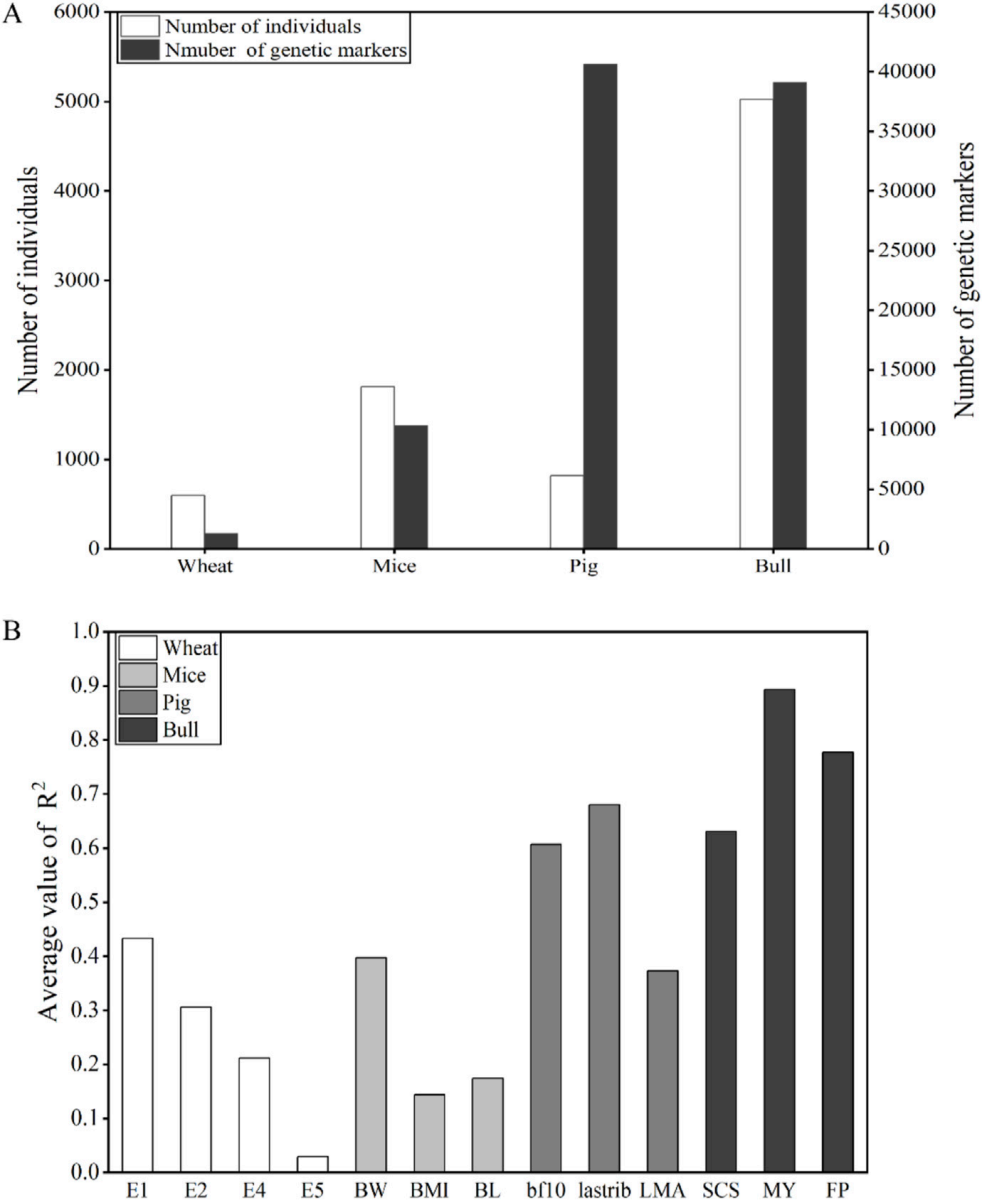
**(A)** Summary of the number of individuals and genetic markers. **(B)** Comparison of average values of $R^{2}$ .

The average values of $R^{2}$ fitted to all the traits/environments of the four species and the summary of population size and genetic markers were compared horizontally, as shown in Figure 5.

### 3.6 Learning curves with bull data

**FIGURE 6:**
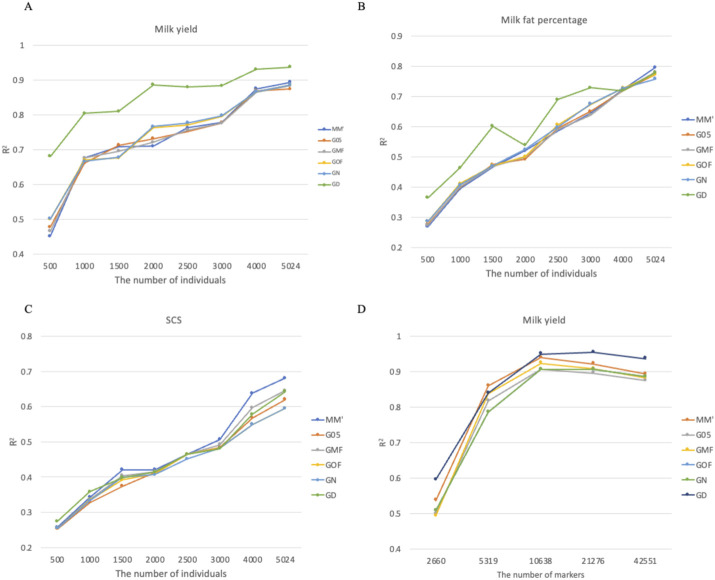
Effects of reference population size and marker density on the accuracy of genomic prediction across different G-matrices in bull data. **(A)** Coefficient of determination for milk yield using different G-matrices and the number of individuals. **(B)** Coefficient of determination for milk fat percentage using different G-matrices and the number of individuals. **(C)** Coefficient of determination for SCS using different G-matrices and the number of individuals. **(D)** Coefficient of determination for milk yield using different G-matrices and the number of markers.

Figures 6A–C showed that reference population size significantly affected prediction accuracy across all three traits. Although accuracy consistently improved as population size increased from 500 to 5,024 individuals, none of the analyses reached a clear plateau, suggesting the potential for further gains with larger reference populations. The choice of the G-matrix showed trait-dependent effects on prediction accuracy. For milk yield, G-matrix selection had a substantial impact at smaller reference sizes (<2,000 individuals), with the GD matrix demonstrating superior performance in these scenarios. For somatic cell score (SCS), prediction accuracy was less sensitive to G-matrix construction methods across all population sizes. Marker density analysis for milk yield revealed that prediction accuracy plateaued at 10,638 markers (1/4 of the full panel), with minimal improvements observed when including additional markers (Figure 6D). The GD matrix consistently outperformed other approaches across most marker density levels, except when the marker density was 1/8 of the full panel.

## 4 Discussion

This study used datasets from four different species to investigate the impact of the G-matrix on the accuracy of genomic prediction. The coefficient of determination, which was calculated according to the sum of squared prediction errors, was used to measure the accuracies of genomic prediction. The results showed that the accuracies of prediction using different G-matrices were not consistent in mice, wheat, and pig data, but they were quite similar in bulls’ data. GD performed the best in all three traits of pigs, while G05, MM′, and GMF performed better than the other methods in wheat. GOF and GN obtained quite similar accuracies in most traits.

In most studies, the accuracy of genomic prediction is assessed using the squared correlation between genomic estimated breeding values (GEBVs) and pseudo phenotypes as the criterion (Ma et al., 2019). In this study, we used $R^{2}$ , calculated as 1 minus the ratio of PRESS to SST. $R^{2}$ has been shown to be equivalent to the squared correlation between GEBVs and the true breeding values (Xu, 2017). This means that the predicted value $\hat{y_{i}}$ explains the ratio of the variance in the observed value $y_{i}$ . The higher the goodness of fit, the higher the degree of explanation of the independent variable to the dependent variable, and the higher the percentage of variation caused by the independent variable to the total variation. It means that the more intensive the observation points are near the regression line, the higher the prediction accuracy of the model.

The genotyping of wheat lines was performed using Diversity Arrays Technology (DArT) (Jaccoud et al., 2001). DArT markers generate binary (0/1) calls that primarily reflect the presence/absence variation (PAV) of genomic fragments rather than allelic dosage information. This means that the technology detects whether a specific genomic fragment is present (1) or absent (0) in a given sample, without differentiating between different allelic states or copy numbers across homologous chromosomes. For example, in hexaploid wheat (AABBDD genome constitution), if a DArT marker is present in at least one of the A sub-genome chromosomes, it will be scored as “1” (present), regardless of whether the marker is present or absent in the B or D sub-genomes.

The optimal method for constructing the G-matrix was not consistent in different species since the population structures were quite different. The single-marker weighted GD matrix could significantly improve the accuracy of prediction for pig traits, while the other five matrices showed no significant difference. The possible reason could be that these traits were affected by different loci, with the hypothesis that the markers were equally informative (Speed and Balding, 2015). The prediction accuracies of the three traits of bulls were high, and there were no significant differences in genomic prediction calculated using the six matrices, which may be due to the large size of the reference population and reliable phenotypes used in the bull data. The comparative analysis of the reference population size effects in bulls revealed patterns consistent with those observed in pig data. Notably, the GD matrix demonstrated superior predictive accuracy for milk yield at smaller reference population sizes (<2,000 individuals), mirroring its enhanced performance for porcine backfat and loin muscle area traits under similar conditions. This consistency across species indicates that the GD approach may be generally preferable for traits influenced by a mix of major and minor genes when working with limited reference data. G05 and GD could slightly improve the prediction accuracy of mouse traits. The mouse population was derived from eight different lines and had undergone more than 50 generations of random mating. According to the results, we inferred that constant a value of 0.5 was suitable for the heterogeneous population. Unlike the other three species, MM′, G05, and GMF could improve the prediction accuracy of wheat traits, and MM′ obtained the highest $R^{2}$ value. The wheat data were from the different lines; the value of 0.5 or without adjustment might be two suitable choices. GOF and GN obtained similar accuracies for almost all the traits, and the reason was that these two methods were similar to the hypothesis that the number of individuals was high.

The size of the reference population and the number of effective SNPs also influence the uncertainty of the estimated effect and variance, whose importance has been demonstrated in many studies (Daetwyler et al., 2008; MacEachern et al., 2009; Rolf et al., 2010; Solberg et al., 2008). The results of the four species were as follows: the average values of $R^{2}$ of bulls were higher than that of pigs and mice, and that of mice was slightly higher than that of wheat. The population size and number of genetic markers of the four species were as follows: bulls had the largest, followed by pigs, then mice, and finally wheat. The bulls’ phenotypes, which were estimated using records from many daughters and other relatives, had high reliability, thereby ensuring high reliability in genomic prediction (Ding et al., 2013). Since the size of the wheat genome is large (International Wheat Genome Sequencing Consortium (IWGSC), 2014) and only 1,279 markers were used in this study, the accuracies of genomic prediction were relatively low. The results of the learning curve using bull data showed a clear pattern: $R^{2}$ increased with the increase in the size of the reference population. However, accuracy reached a plateau at 10,638 markers (1/4 of full panel). The comprehensive results showed that the reference population size exhibited substantially greater impact on prediction accuracy than marker density.

The higher the heritability, the more the phenotype is influenced by gene control, and the more accurately the regression model predicted. However, the order of different methods for the traits of the same species was similar. The three pig traits used in this study had moderate to high heritability (Cabling et al., 2015). The accuracies of genomic prediction for two backfat traits were higher than that for LMA trait. SCS is affected by many small effect loci, and the $R^{2}$ value is lowest in bulls. MY is influenced by few moderate effect loci, and the $R^{2}$ value is the highest. FP is influenced by one major gene and many small effector loci, and the $R^{2}$ value is very close to MY. The results showed that major genes could also affect the prediction accuracy of the model. However, the differences among the three traits are not significant. It also shows that when the number of reference populations and the number of effective genetic markers reach a certain level, the G-matrix of these six transformations has little influence on the prediction accuracy of the GBLUP model. It is worth mentioning that G05 significantly improved the prediction accuracy of BW and GD significantly improved the accuracy of BMI among low heritability traits such as body weight and body length related to obesity in mice. The mouse population came from multiple full sibling families with a high coefficient of inbreeding, and body weight and body length were low heritability traits controlled by multiple genes with a small effect. Therefore, for the three traits of mice in the study, G05 can improve the prediction accuracy of the genomic prediction model (Zhang et al., 2019).

This result is also reflected in wheat yield projects. The accuracies of the G-matrix, obtained using the average allele frequency and MAF of 0.5, were higher than those of the other methods. This is related to the strong influence of the plant environment, unknown allele frequency, and a small number of reference populations and SNPs. The results indicated that the optimal method for constructing the G-matrix in genomic prediction was influenced by the population structure strongly rather than the genetic character of the traits. GOF and GN obtained similar accuracies for almost all the traits, and the reason was that these two methods were similar with the hypothesis that the number of individuals was high.

After deleting SNPs with MAF less than 0.05, the number of SNPs in pigs and bulls was 40,653 and 39,117, respectively. The changes in population numbers of wheat, mice, and bulls are consistent with the changes in the number of genetic markers. The number of individuals and genetic markers in bulls is abundant. The population size and genetic marker quantity of wheat and mice were lower. Pigs have a relatively small number of individuals but the largest number of genetic markers. The average values of $R^{2}$ of wheat and mice were both lower than 0.5, indicating low prediction accuracy. Except for the LMA of pigs, the average values of $R^{2}$ of pigs and bulls were all greater than 0.6, indicating high prediction accuracy. Bulls had the highest predictive accuracy among the four species.

A limitation to this study is the consideration of additive effects only. Dominant variance components (DeVogel et al., 2021) could also be considered in the future if the traits were also affected by dominant effects.

## 5 Conclusion

Different G-matrix construction methods exhibit significant differences in the accuracy of prediction, especially for pig and wheat data. The performance of different G-matrix construction methods in various species was not consistent. Population structure could be considered one of the important factors for choosing the method of constructing the G-matrix. When the reference population and genetic marker density reached a certain scale, the six matrices in this study had little influence on the prediction accuracy of the GBLUP model.

## Data Availability

The data presented in the study are publicly available from the following sources. Mice and wheat datasets were obtained from the BGLR reference manual (https://cran.r-project.org/web/packages/BGLR/BGLR.pdf) of R software (de los Campos et al., 2009). Bull dataset was provided by Vereinigte Informationssysteme Tierhaltung w.V. Pig data was available from the previous published work of Fan et al., 2011.
