# Supplementary material for: Impact of different genomic relationship matrix construction methods on the accuracy of genomic prediction in different species
Source: Front Genet. 2025 May 2;16:1576248. doi: 10.3389/fgene.2025.1576248 (PMC12082045; doi:10.3389/fgene.2025.1576248)
Supplement: Supplementary file 2 [file DataSheet1.docx]

mixedBlup<-function(x,y,aa,method="REML",h2=NULL){

loglike<-function(theta){

lambda<-exp(theta)

logdt<-sum(log(lambda*delta+1))

h<-1/(lambda*delta+1)

yy<-sum(yu*h*yu)

yx<-matrix(0,s,1)

xx<-matrix(0,s,s)

for(i in 1:s){

yx[i]<-sum(yu*h*xu[,i])

for(j in 1:s){

xx[i,j]<-sum(xu[,i]*h*xu[,j])

}

}

if(method=="REML"){

loglike<- -0.5*logdt-0.5*(n-s)*log(yy-t(yx)%*%solve(xx)%*%yx)-0.5*log(det(xx))

} else {

loglike<- -0.5*logdt-0.5*n*log(yy-t(yx)%*%solve(xx)%*%yx)

}

return(-loglike)

}

fixed<-function(lambda){

h<-1/(lambda*delta+1)

yy<-sum(yu*h*yu)

yx<-matrix(0,s,1)

xx<-matrix(0,s,s)

for(i in 1:s){

yx[i]<-sum(yu*h*xu[,i])

for(j in 1:s){

xx[i,j]<-sum(xu[,i]*h*xu[,j])

}

}

beta<-solve(xx,yx)

if(method=="REML"){

sigma2<-(yy-t(yx)%*%solve(xx)%*%yx)/(n-s)

} else {

sigma2<-(yy-t(yx)%*%solve(xx)%*%yx)/n

}

var<-diag(solve(xx)*drop(sigma2))

stderr<-sqrt(var)

return(c(beta,stderr,sigma2))

}

blup<-function(par,x,y,aa){

s<-length(par)

k1<-which(!is.na(y))

k2<-1:length(y)

a11<-aa[k1,k1]

a21<-aa[k2,k1]

a22<-aa[k2,k2]

y1<-as.matrix(y[k1])

x1<-as.matrix(x[k1,])

beta<-matrix(par[1:(s-2)],(s-2),1)

va<-par[s-1]

ve<-par[s]

v<-a11*va+diag(length(y1))*ve

random<-va*a21%*%solve(v)%*%(y1-x1%*%beta)

fixed<-x%*%beta

ypred<-random+fixed

vpred<-diag(va*(a22-va*a21%*%solve(v)%*%t(a21)))

stderr<-sqrt(abs(vpred))

return(data.frame(y,fixed,random,ypred,stderr))

}

k1<-which(!is.na(y))

k2<-1:length(y)

a11<-aa[k1,k1]

a21<-aa[k2,k1]

y1<-as.matrix(y[k1])

x1<-as.matrix(x[k1,])

n<-nrow(y1)

qq<-eigen(a11,symmetric=T)

delta<-qq[[1]]

uu<-qq[[2]]

s<-ncol(x1)

yu<-t(uu)%*%y1

xu<-t(uu)%*%x1

if(is.null(h2)){

theta<-0

parm<-optim(par=theta,fn=loglike,NULL,hessian = FALSE, method="L-BFGS-B",lower=-20,upper=20)

lambda<-exp(parm$par)

conv<-parm$convergence

fn1<-parm$value

fn0<-loglike(-Inf)

lrt<-2*(fn0-fn1)

parmfix<-fixed(lambda)

beta<-parmfix[1:s]

stderr<-parmfix[(s+1):(2*s)]

ve<-parmfix[2*s+1]

lod<-lrt/4.61

p_value<-1-pchisq(lrt,1)

va<-lambda*ve

h2<-va/(va+ve)

par<-data.frame(method,beta,stderr,va,ve,lambda,h2,conv,fn1,fn0,lrt,lod,p_value)

} else {

lambda<-h2/(1-h2)

parmfix<-fixed(lambda)

beta<-parmfix[1:s]

stderr<-parmfix[(s+1):(2*s)]

ve<-parmfix[2*s+1]

va<-lambda*ve

par<-data.frame(method,beta,stderr,va,ve,lambda,h2)

}

blup<-blup(par=c(beta,va,ve),x,y,aa)

return(list(par,blup))

}
